# A population-based propensity-matched study of regional dissections in patients with metastatic osteosarcoma

**DOI:** 10.1186/s13018-020-01592-y

**Published:** 2020-03-13

**Authors:** Wenjuan Wang, Hongzhi Ding, Zhenyu Sun, Chen Jin, Yanhui Zhu, Xiang Wang

**Affiliations:** 1 Shanghai Key Laboratory of Orthopaedic Implants, Shanghai Ninth People’s Hospital, Shanghai Jiao Tong University School of Medicine, Shanghai, China; 2 Department of Orthopaedic Surgery, Shanghai Ninth People’s Hospital, Shanghai Jiao Tong University School of Medicine, Shanghai, China

**Keywords:** metastatic osteosarcoma, regional dissection, primary tumours, SEER, overall survival, cancer-specific survival

## Abstract

**Background:**

The survival rates of patients with metastatic osteosarcoma are poor, and the prognosis is closely related to the choice of treatment, especially surgery. This study aimed to evaluate the survival outcomes of patients with metastatic osteosarcoma undergoing regional dissections.

**Methods:**

We collected data on patients with metastatic osteosarcoma between 2004 and 2014 from the Surveillance, Epidemiology, and End Results (SEER) database. Kaplan–Meier curves were used to compare overall survival (OS) and cancer-specific survival (CSS), while univariate and multivariate Cox regression analyses were used to evaluate outcomes. Propensity score matching (PSM) was used to minimize the effects of confounding factors.

**Results:**

The SEER database had records of 2768 patients diagnosed with osteosarcoma, of whom 398 were included in our study. Of the included patients, 116 (29.15%) underwent regional dissections, while 282 (70.85%) underwent non-regional dissections. The univariate and multivariate Cox regression analyses, prior to PSM, showed that OS (hazard ratio (HR): 0.34, 95% confidence interval (CI): 0.26–0.44, P<0.001 and HR: 0.47, 95% CI: 0.35–0.64, P<0.001, respectively) and CSS (HR: 0.33, 95% CI: 0.25–0.43, P<0.001 and HR: 0.46, 95% CI: 0.34–0.63, P<0.001, respectively) were better in patients who underwent regional dissections than those who underwent non-regional dissections. Compared with non-regional dissections, regional dissections, which included both primary tumour resection (PTR) and primary tumour and metastatic site resection (PTMR), were associated with better OS (P<0.001) and CSS (P<0.001) . However, the survival outcomes following PTR and PTMR showed no significant difference. After PSM, patients in the regional dissection group still had a higher OS (P<0.001) and CSS (P<0.001) than those in the non-regional dissection group.

**Conclusions:**

Compared with non-regional dissection, regional dissection resulted in better survival in patients with metastatic osteosarcoma.

## Introduction

Osteosarcoma is the most common primary malignant bone tumour in children and adolescents [1]. It is characterized by rapid progression, early pulmonary metastasis, poor prognosis and recurrence [2–4]. While the 5-year survival rate of patients with osteosarcoma is approximately 65%, that of patients with metastatic osteosarcoma is only 25% [5]. The poor prognosis in patients with metastatic osteosarcoma highlights the need for a more effective therapy to treat both primary and metastatic tumours and to improve the patients’ quality of life and survival rates. Though advanced chemotherapy has the potential to increase the overall survival (OS), it is, however, not as effective by itself in the absence of surgical resection [6].

While the survival factors associated with metastatic osteosarcoma are complex and controversial, surgical resection is widely accepted as a beneficial treatment method. Some retrospective studies have reported the benefits of primary tumour resection (PTR) and primary tumour and metastatic site resection (PTMR) [7–9]. In general, surgical resection, combined with chemotherapy, has been shown to result in better prognosis [10, 11]. However, whether surgery is beneficial for stage IV osteosarcoma patients with extensive metastases remains unclear. There have been studies that have favoured non-resection therapies, such as chemotherapy or radiotherapy, for patients with multiple unresectable metastases [2, 12]. Surgical resection is not always feasible due to challenges such as large tumours, various sites of origin, poor physical condition, and various complications after resection. In particular, when the expected survival time is less than three months after surgery, surgical resection is not an optimal alternative. The choice of surgical resection in patients with metastatic osteosarcoma has, therefore, not been widely reported.

Despite these controversies, we used the available data on patients with metastatic osteosarcoma in the United States from the Surveillance, Epidemiology, and End Results (SEER) database, to evaluate the OS and cancer-specific survival (CSS) and to determine if regional dissections are beneficial for these patients. We believe that our findings would help with the selection of the right surgical treatment and thereby improve the prognosis for metastatic osteosarcoma.

## Methods

### Data source

The SEER database supported by the National Cancer Institute includes data on patients with metastatic osteosarcoma such as the patient demographics, treatments, and survival times. For our analysis, we collected data from 18 population-based cancer registries of the SEER database (1973–2014 dataset), accounting for 30% of the US population.

### Inclusion and exclusion criteria

The patients included in the study (1) had osteosarcoma, which was the only cancer that was included, (2) were diagnosed from 2004 to 2014, (3) had a survival time of > 3 months, (4) were eligible for a specific treatment of primary tumours, and (5) had other synchronous cancers along with osteosarcoma. Patients not diagnosed with stage IV osteosarcoma were excluded from the study.

### Institutional Review Board approval

Data used in this study were obtained from the SEER database. The private SEER ID (13130-Nov 2017) was used to support our analysis. All procedures performed in studies involving human participants were in accordance with the ethical standards of the Institutional and/or National Research Committee and with the 1964 Helsinki declaration and its later amendments or comparable ethical standards.

### Propensity score matching

We used propensity score matching (PSM) to avoid the use of unbalanced basic patient variables and thereby avert a selection bias. First, a logistic regression model was set up, and regional dissection was regarded as the dependent variable. Table 1 summarizes the baseline characteristics of the included patients. Next, patients who underwent regional dissections were matched with those who had not undergone regional dissections based on the calculated scores with a greedy algorithm of the nearest matched neighbour at a fixed ratio of 1:1. After matching, we checked if these covariates were balanced on the basis of absolute values. An absolute value < 0.1 was indicative of a good balance between the two groups.

**Table 1:** Characteristics of patients with metastatic osteosarcoma included in this study

Characteristics	Total	Regional dissection	Non-regional dissection
	398 (100%)	116 (29.15%)	282 (70.85%)
Sex			
Female	160 (40.20%)	51 (43.97%)	109 (38.65%)
Male	238 (59.80%)	65 (56.03%)	173 (61.35%)
Race			
White	307 (77.14%)	94 (81.03%)	213 (75.53%)
Black	59 (14.82%)	12 (10.34%)	47 (16.67%)
Other race	32 (8.04%)	10 (8.62%)	22 (7.80%)
Age			
<18	221 (55.53%)	38 (32.76%)	183 (64.89%)
18-65	147 (36.93%)	62 (53.45%)	85 (30.14%)
>=65	30 (7.54%)	16 (13.79%)	14 (4.96%)
Grade			
Well/moderate	11 (2.76%)	5 (4.31%)	6 (2.13%)
Poorly/undifferentiated	290 (72.86%)	76 (65.52%)	214 (75.89%)
Unknown	97 (24.37%)	35 (30.17%)	62 (21.99%)
T Stage			
T1	67 (16.83%)	21 (18.10%)	46 (16.31%)
T2	220 (55.28%)	44 (37.93%)	176 (62.41%)
T3	26 (6.53%)	9 (7.76%)	17 (6.03%)
Tx	85 (21.36%)	42 (36.21%)	43 (15.25%)
Chemotherapy			
No/unknown	26 (6.53%)	12 (10.34%)	14 (4.96%)
Yes	372 (93.47%)	104 (89.66%)	268 (95.04%)
Radiation			
No/unknown	334 (83.92%)	79 (68.10%)	255 (90.43%)
Yes	64 (16.08%)	37 (31.90%)	27 (9.57%)
Location			
Extremity	326 (81.91%)	71 (61.21%)	255 (90.43%)
Other	72 (18.09%)	45 (38.79%)	27 (9.57%)

### Statistical analyses

In our analysis, OS and CSS were regarded as the standard results. The Kaplan–Meier plot was used to estimate and plot the survival curves. The curves of the regional dissection and non-regional dissection groups were compared using the log-rank test. Variables with P values <0.05 in the univariate analysis were subsequently used in the multivariate analysis. A Cox proportional hazards model was established before matching. The outcomes were presented as 95% confidence intervals (CIs) and hazard ratios (HRs). All analyses were two-sided and were performed using the SPSS software (version 23.0; IBM, NY), and P < 0.05 was considered statistically significant.

## Results

### Patient characteristics

**Fig. 1:**
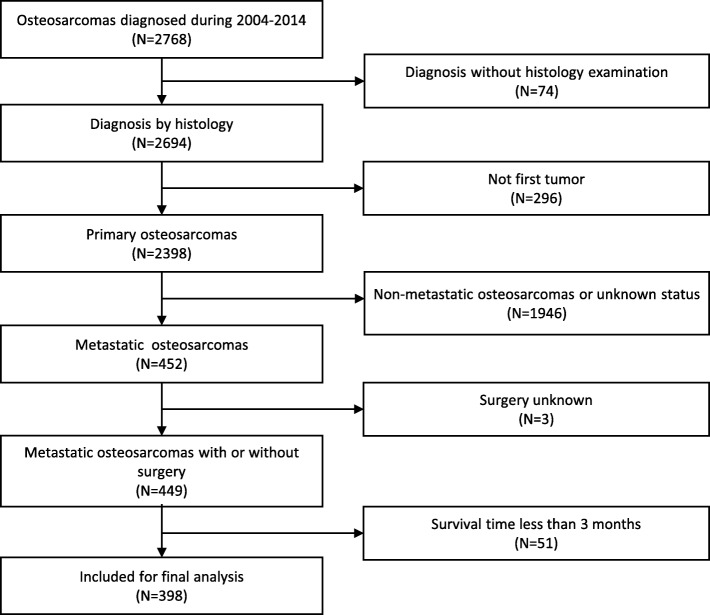
Flow chart showing the patient selection process

The SEER database had records of 2,768 patients with osteosarcomas between 2004 and 2014. On the basis of the inclusion and exclusion criteria, 398 out of these 2,768 patients were included in our analysis. The process of patient selection is shown in Fig. 1. The baseline characteristics of the 398 patients included in the study are shown in Table 1. Approximately 59.80% of the patients were men, and 77.14% of them belonged to the white race. In total, 116 patients underwent regional dissections, and 282 underwent non-regional dissections. Furthermore, approximately 93.47% and 16.08% of the patients underwent chemotherapy and radiation, respectively.

### Survival analyses before population matching

**Fig. 2:**
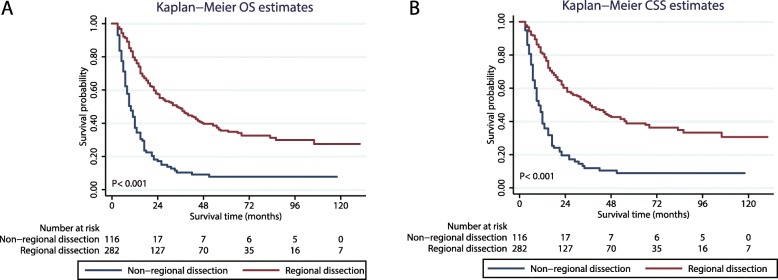
Kaplan–Meier survival analysis. Shown are curves comparing the (**a**) OS and (**b**) CSS in metastatic osteosarcoma patients who underwent regional dissection and those who did not undergo regional dissection. OS: overall survival; CSS: cause-specific survival

The OS in patients who underwent different treatments (non-regional dissection vs regional dissection) was analysed using the Kaplan-Meier method (Fig. 2a). The curves showed that prior to PSM, the OS was better in the regional dissection group than in the non-regional dissection group (P < 0.001) (Fig. 2). Patients who underwent regional dissection were also found to have a better CSS than those who did not (P < 0.001) (Fig. 2b).

The reliability of the survival results was verified by univariate analysis. We found that patients in the regional dissection group had better OS (HR: 0.34, 95% CI: 0.26-0.44, P < 0.001) and CSS (HR: 0.33, 95% CI: 0.25-0.43, P < 0.001) those in the non-regional dissection group (Table 2). In addition, radiation and location factors were found to influence the OS and CSS (Table 2). Interestingly, the multivariate Cox regression analysis revealed that patients who underwent lymph node dissections had a higher OS (HR: 0.47, 95% CI: 0.35-0.64, P < 0.001) and CSS (HR: 0.46, 95% CI: 0.34-0.63, P < 0.001) (Table 3). Based on the P values, age at diagnosis was found to be a potential risk factor for the development of primary metastatic osteosarcoma (Table 3).

**Table 2:** Univariate Cox regression analysis for evaluating the influence of Regional dissection on survival of patients with primary metastatic osteosarcoma in SEER database

Characteristics	OS		CSS	
	HR (95% CI)	P	HR (95% CI)	P
Treatment				
Non-regional dissection	Reference		Reference	
Regional dissection	0.34 (0.26-0.44)	<0.001	0.33 (0.25-0.43)	<0.001
Sex				
Female	Reference		Reference	
Male	1.00 (0.78-1.29)	0.980	1.03 (0.79-1.33)	0.851
Race				
White	Reference		Reference	
Black	0.77 (0.53-1.12)	0.170	0.72 (0.48-1.08)	0.114
Other race	1.29 (0.82-2.02)	0.268	1.39 (0.89-2.18)	0.152
Age				
<18	Reference		Reference	
18-65	2.16 (1.66-2.80)	<0.001	2.14 (1.63-2.81)	<0.001
>=65	4.11 (2.65-6.38)	<0.001	4.48 (2.88-6.98)	<0.001
Grade				
Well/moderate	Reference		Reference	
Poorly/undifferentiated	0.53 (0.28-1.00)	0.049	0.48 (0.25-0.91)	0.024
Unknown	0.65 (0.34-1.27)	0.212	0.61 (0.31-1.19)	0.148
T Stage				
T1	Reference		Reference	
T2	0.97 (0.68-1.39)	0.884	0.97 (0.67-1.39)	0.855
T3	1.48 (0.84-2.59)	0.171	1.56 (0.88-2.75)	0.121
Tx	1.26 (0.84-1.88)	0.269	1.13 (0.74-1.72)	0.584
Chemotherapy				
No/unknown	Reference		Reference	
Yes	0.53 (0.34-0.83)	0.005	0.49 (0.31-0.76)	0.001
Radiation				
No/unknown	Reference		Reference	
Yes	2.20 (1.62-2.99)	<0.001	2.20 (1.60-3.03)	<0.001
Location				
Extremity	Reference		Reference	
Other	2.53 (1.89-3.41)	<0.001	2.50 (1.84-3.41)	<0.001

Abbreviation: SEER=Surveillance, Epidemiology and End Results; OS=Overall survival; CSS=Cause-specific survival

**Table 3:** Multivariate Cox regression analysis for evaluating the influence of Regional dissection on survival of patients with primary metastatic osteosarcoma in SEER database

Characteristics	OS		CSS	
	HR (95% CI)	P	HR (95% CI)	P
Treatment				
Non-regional dissection	Reference		Reference	
Regional dissection	0.47 (0.35-0.64)	<0.001	0.46 (0.34-0.63)	<0.001
Age				
<18	Reference		Reference	
18-65	1.79 (1.36-2.36)	<0.001	1.77 (1.32-2.36)	<0.001
>=65	2.39 (1.40-4.06)	0.001	2.54 (1.48-4.36)	0.001
Grade				
Well/moderate	Reference		Reference	
Poorly/undifferentiated	0.86 (0.44-1.65)	0.644	0.80 (0.41-1.54)	0.496
Unknown	0.80 (0.41-1.59)	0.531	0.76 (0.38-1.50)	0.423
Chemotherapy				
No/unknown	Reference		Reference	
Yes	0.98 (0.57-1.67)	0.939	0.93 (0.54-1.59)	0.779
Radiation				
No/unknown	Reference		Reference	
Yes	1.25 (0.88-1.78)	0.210	1.22 (0.85-1.76)	0.285
Location				
Extremity	Reference		Reference	
Other	1.28 (0.90-1.83)	0.166	1.24 (0.86-1.79)	0.253

Abbreviation: SEER=Surveillance, Epidemiology and End Results; OS=Overall survival; CSS=Cause-specific survival

**Fig. 3:**
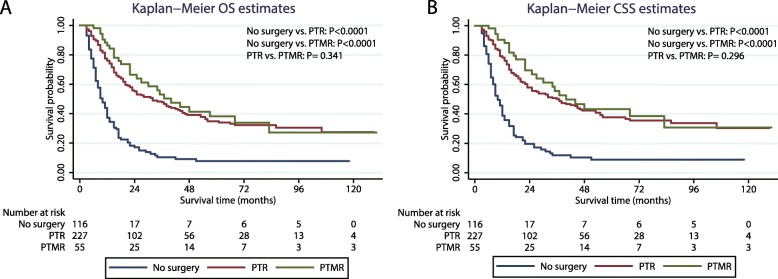
Kaplan–Meier survival analysis. Shown are curves comparing the (**a**) OS and (**b**) CSS between the non-regional dissection group, PTR group, and PTMR group. OS: overall survival; CSS: cause-specific survival; PTR: primary tumour resection; PTMR: primary tumour and metastatic site resection

To identify the specific therapies that effectively improved survival rates, we divided the regional dissection group into two subgroups: PTR and PTMR. As shown in Fig. 3, both the subgroups had better survival (OS or CSS) than the non-regional dissection group (P < 0.001). However, no significant difference was observed in the survival outcomes between the PTR and PTMR subgroups.

### Survival analyses after population matching

**Fig. 4:**
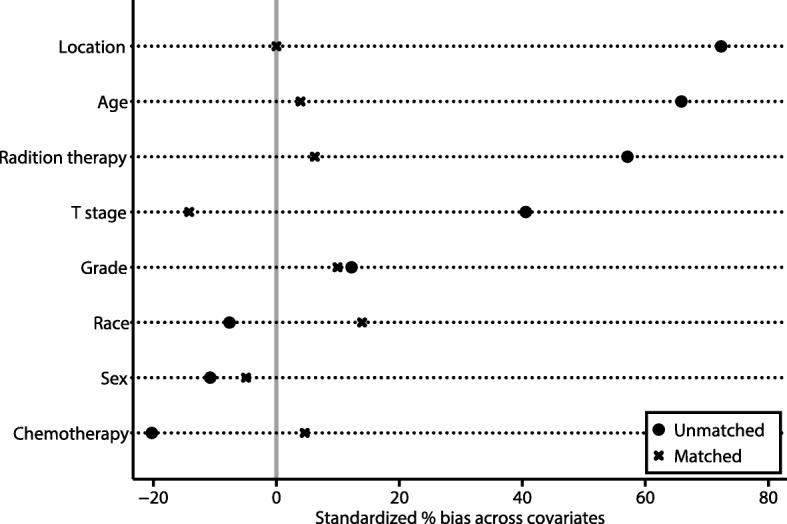
Standardised bias test. The standardised bias (%) across covariates before and after propensity score matching show that the candidate covariates were well matched

**Fig. 5:**
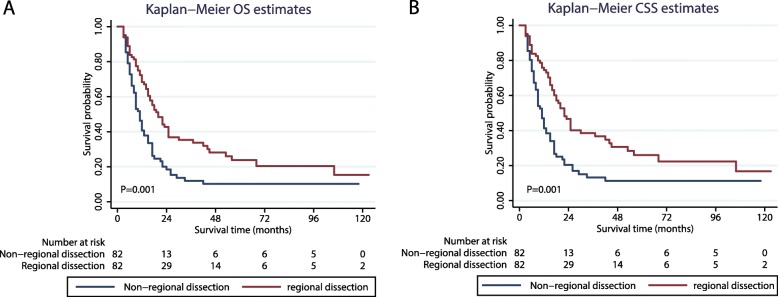
Kaplan–Meier survival analysis. Shown are the curves for (**a**) OS and (**b**) CSS based on the primary tumour local treatment status in the matched population. OS: overall survival; CSS: cause-specific survival

Several confounding factors were eliminated by PSM. The results of the test for standardised bias across covariates before and after PSM at a 1:1 fixed ratio showed that candidate covariates were well matched (Fig. 4). The Kaplan-Meier curves for OS and CSS showed that patients who underwent regional dissections had better OS and CSS than those who did not undergo regional dissections (Fig. 5).

## Discussion

Our results showed that regional dissection played an essential role in improving the prognosis in patients with metastatic osteosarcoma. Moreover, patients who underwent PTR or PTMR had higher survival rates than those who did not undergo regional resection.

To the best of our knowledge, this is the first study to report an association between regional dissections at the primary site and better survival in patients with metastatic osteosarcoma based on data from the SEER database. While some previous studies have evaluated the available treatments for metastatic osteosarcoma [6, 13–15] , they are considered to be outdated and inadequate, given the development of various new diagnostic and surgical technologies. Hence, updating this data is important to make it relevant. Our analysis was based on data from 2004 to 2014 and used novel methods, such as PSM to reduce the effects of confounding factors.

Our finding that regional dissection of metastatic osteosarcoma can improve the OS in patients is consistent with previous findings [6]. Kempf*-*Bielack et al. [16] reported improvements in patient survival following surgical treatments. Patients who did not receive any surgical interventions were at a higher risk of mortality than those who underwent complete surgical dissection of all the detected tumours [17]. Although the relationship between surgical dissection and better survival remained unclear, the phenomenon was supported by some theories. The Halsted theory revealed that tumours spread in an orderly pattern and extended in an adjacent fashion from the primary tumour through the lymphatics to the lymph nodes and then to distant sites [18]. In contrast, other studies have suggested that the growth and metastasis of primary tumours may be complicated and multidirectional [19, 20]. Kim et al. showed that circulating tumour cells (CTCs) colonised their site of origin, which is known as “tumour self-seeding” [20]. Furthermore, the CTCs, which accelerate metastatic tumour formation [21], can alter the microenvironment, making it more favourable for tumour growth without further adaptation [19]. Tumour self-seeding by CTCs has been reported in osteosarcomas [22] , and therefore, primary tumours should be excised to reduce the CTCs. We found that compared to the non-regional dissection of metastatic osteosarcomas, the regional dissection of primary tumours may be a more appropriate approach to promote survival.

The Cooperative German-Austrian-Swiss Osteosarcoma Study Group showed that concomitant regional dissection of primary tumours can improve the effectiveness of chemotherapy in patients with metastatic osteosarcoma [23] . Some other studies suggested that surgical resection of all detectable neoplasms contributed to better prognosis [6, 24]. Surgical dissection of all metastatic sites has, therefore, been recommended for patients with metastatic osteosarcoma [25, 26]. Studies have also indicated that the aggressive resection of tumours, both primary and metastatic, improves the patient’s response to chemotherapy [27–29]. In this study, we demonstrated that patients who underwent PTR or PTMR had better survival than those who did not undergo regional resection. However, we found no significant difference in the survival outcomes following PTR versus PTMR. Therefore, the choice of surgical method depends on individual circumstances. For instance, it is not advisable for a metastatic osteosarcoma patient with poor physical condition to undergo complete surgical dissection of all detected tumours if the survival time following the dissection is comparable to that of patients undergoing PTR. This is because a complete surgery, such as PTMR, results in more complications and pain that can last a lifetime. Furthermore, PTR is a less difficult procedure for the doctor to perform and has a higher success rate than PTMR. Other studies have also proposed that patients with diseases in multiple sites should not undergo complete surgical dissection because of poor prognosis [6]. Compared to PTMR, PTR, therefore, is more appropriate for such patients and is associated with similar survival rates. Studies have shown that palliative surgical resection can help alleviate symptoms, provide pain relief, and improve survival outcomes [30].

We did not include the quality of life after surgical resection in our analysis. However, we suggest that it should be taken into account since surgery-related complications tend to have adverse effects on the quality of life. In patients with comparable survival outcomes, improvement in the quality of life is important [31]. For comprehensive care, it is also important to consider patient preferences and hospital costs before implementing treatments.

This study has several limitations. Some variates such as response to chemotherapy and the general state of health, which are related to survival, were not recorded in the SEER database. The SEER database also lacked clarification regarding specific procedures used for surgical resection such as limb-sparing surgery and amputation, which may have led to differences in the quality of life. Moreover, there could have been unobserved confounders that were probably not adjusted for in this analysis. For example, doctors may prefer a certain surgical treatment for patients with better prognosis. This selection bias may have a potential effect on the outcome of resection and might make results look better than they actually were. If the underlying prognostic variables were known, PSM could be used to reduce the effect of these biases. Despite these limitations, we have reduced the biases and errors as far as possible by using effective measures such as Kaplan–Meier plot before and after PSM, as well as univariate and multivariate analyses.

## Conclusions

In conclusion, patients in the regional dissection group, including the PTR and PTMR subgroups, had better OS and CSS than those in the non-regional dissection group based on the case-matched analysis. Furthermore, PTR was more appropriate than PTMR for patients with metastatic osteosarcoma who had comparable survival rates. However, further clinical studies are required to confirm these findings.

## Data Availability

Data used in this study were obtained from the SEER database. The private SEER ID (13130-Nov 2017) was used to support our analysis.

## Abbreviations

CI: Confidence interval
CSS: Cancer-specific survival
CTCs: circulating tumour cells
HR: Hazard ratio
OS: Overall survival
PSM: Propensity score matching
PTMR: Primary tumour and metastatic site resection
PTR: Primary tumour resection
SEER: Surveillance, Epidemiology, and End Results
SPSS: Statistical package for the social sciences
